# Gestational Hypothyroxinemia Affects Its Offspring With a Reduced Suppressive Capacity Impairing the Outcome of the Experimental Autoimmune Encephalomyelitis

**DOI:** 10.3389/fimmu.2018.01257

**Published:** 2018-06-06

**Authors:** Henny Haensgen, Eduardo Albornoz, María C. Opazo, Katherinne Bugueño, Evelyn Liliana Jara Fernández, Rebecca Binzberger, Tomás Rivero-Castillo, Luis F. Venegas Salas, Felipe Simon, Claudio Cabello-Verrugio, Alvaro A. Elorza, Alexis M. Kalergis, Susan M. Bueno, Claudia A. Riedel

**Affiliations:** ^1^Departamento de Ciencias Biológicas, Facultad de Ciencias de la Vida, Universidad Andrés Bello, Santiago, Chile; ^2^Millennium Institute on Immunology and Immunotherapy, Santiago, Chile; ^3^Departamento de Genética Molecular y Microbiología, Facultad de Ciencias Biológicas, Pontificia Universidad Católica de Chile, Santiago, Chile; ^4^Medizinishen Fakultät, Eberhard Karls Universität, Tübingen, Germany; ^5^Departamento Biomédico, Facultad de Ciencias de la Salud, Universidad de Antofagasta, Antofagasta, Chile; ^6^Centro de Investigaciones Biomédicas, Facultad de Ciencias de la Vida y Facultad de Medicina, Universidad Andrés Bello, Santiago, Chile; ^7^Departamento de Endocrinología, Escuela de Medicina, Facultad de Medicina, Pontificia Universidad Católica de Chile, Santiago, Chile

**Keywords:** hypothyroxinemia, T regulatory cells, multiple sclerosis, pregnancy, experimental autoimmune encephalomyelitis

## Abstract

Hypothyroxinemia (Hpx) is a thyroid hormone deficiency (THD) condition highly frequent during pregnancy, which although asymptomatic for the mother, it can impair the cognitive function of the offspring. Previous studies have shown that maternal hypothyroidism increases the severity of experimental autoimmune encephalomyelitis (EAE), an autoimmune disease model for multiple sclerosis (MS). Here, we analyzed the immune response after EAE induction in the adult offspring gestated in Hpx. Mice gestated in Hpx showed an early appearance of EAE symptoms and the increase of all parameters of the disease such as: the pathological score, spinal cord demyelination, and immune cell infiltration in comparison to the adult offspring gestated in euthyroidism. Isolated CD4^+^CD25^+^ T cells from spleen of the offspring gestated in Hpx that suffer EAE showed reduced capacity to suppress proliferation of effector T cells (T_Eff_) after being stimulated with anti-CD3 and anti-CD28 antibodies. Moreover, adoptive transfer experiments of CD4^+^CD25^+^ T cells from the offspring gestated in Hpx suffering EAE to mice that were induced with EAE showed that the receptor mice suffer more intense EAE pathological score. Even though, no significant differences were detected in the frequency of T_reg_ cells and IL-10 content in the blood, spleen, and brain between mice gestated in Hpx or euthyroidism, T cells CD4^+^CD25^+^ from spleen have reduced capacity to differentiate *in vitro* to T_reg_ and to produce IL-10. Thus, our data support the notion that maternal Hpx can imprint the immune response of the offspring suffering EAE probably due to a reduced capacity to trigger suppression. Such “imprints” on the immune system could contribute to explaining as to why adult offspring gestated in Hpx suffer earlier and more intense EAE.

## Introduction

Maternal thyroid hormones (THs), 3,5,3′-l-tri-iodotironine (T_3_), and 3,5,3′5′-l-tetraiodo-tironine (T_4_) are essential for the proper development of the central nervous system (CNS), the lungs, skeletal muscles, and bones (1, 2). It has been widely shown in humans and other mammals that maternal thyroid hormone deficiencies (THD), such as hypothyroidism or hypothyroxinemia (Hpx) during gestation can be hazardous for the future cognitive performance of the offspring (3–5). Hpx is highly frequent condition in women with a prevalence fluctuating between 1 and 2% in iodine sufficient communities (6) and considered to be 100–200 times more frequent than congenital hypothyroidism (7). Clinically, low blood levels of T_4_ with normal levels of T_3_ and thyroid stimulating hormone (TSH) are characteristic of this condition. Hpx is asymptomatic for the mother, because the levels of T_3_ are in the normal range and T_3_ is the biologically active TH responsible for most of the effects that THs have on the body. Conversely, the effects of Hpx on the offspring can be significantly detrimental (8, 9) because up to the 11th week of gestation the hypothalamus–hypophysis axis and the thyroid gland of the fetus remain immature and unable to synthesize THs (10). Maternal T_4_ is the only TH able to cross the placenta, then the mother provide T_4_ to the fetus, whose tissues catalyze the transformation into the active T_3_ (11). It has been strongly reported that conditions associated with low T_4_ like maternal hypothyroidism or maternal Hpx caused cognition impairment (7, 12). However, only a few reports have explored the effect of maternal THD during gestation on the immune system of the offspring. It has been reported that the induction of neonatal hypothyroidism in rats caused that their offspring develop reduced numbers of splenic and thymic B and T cells (13). These effects were only transient, as all values returned to normal during the adulthood (13). Also, a transient increase in the frequency of CD4^+^CD25^+^ T cells was observed in the offspring of rats gestated in hypothyroidism; however, after 3 months these values were similar to control rats (14). These facts led to think that the immune system is not altered during gestation by maternal THs. Conversely, Albornoz et al., by using an experimental animal model for multiple sclerosis (MS) named experimental autoimmune encephalomyelitis (EAE) (15), showed that female offspring gestated in hypothyroidism has higher susceptibility to develop EAE compared to the offspring gestated in euthyroidism (16). Thus, it is possible that THD during gestation could work as a risk factor for the adult offspring to suffer this inflammatory disease. To understand the consequences of gestational THD over the offspring immune system, here we have evaluated the effects of gestational Hpx over the suppressive capacity of the immune response of mice suffering EAE. Noteworthy, is to emphasize that this study focuses in the imprinting of gestational Hpx and not gestational hypothyroidism. The reason for that relies that Hpx in pregnancy is 200 times more frequent than it is hypothyroidism (17) and that this condition goes unnoticed by the patient or clinicians because it is absent of symptoms (9). The results of this work showed that the adult offspring gestated in Hpx had an earlier onset and high severity of EAE compared to the progeny gestated in euthyroidism. This observation correlated with a significant increase of spinal cord demyelination and infiltration of CD4^+^ and CD8^+^ T cells in the offspring gestated in Hpx. CD4^+^CD25^+^ T cells isolated from spleen of the offspring gestated in Hpx showed reduced suppressive capacity both *in vitro* and in adoptive transfer experiments to naïve recipient mice gestated in Hpx that suffers EAE. Moreover, CD4^+^CD25^−^ T cells after being *in vitro* stimulated with anti-CD3 and anti-CD28 antibodies in an incubation medium to induce T_reg_ have less capacity to express FOXP3 and IL-10. This study supports the notion that gestational Hpx imprints a reduced capacity in CD4^+^ T cells to trigger suppression in the offspring and this could increase the severity of an inflammatory disease such as EAE.

## Materials and Methods

### Mice

C57BL/6 mice (The Jackson Laboratory, Bar Harbor, ME, USA) were maintained in a germ-free animal facility center at the Universidad Andrés Bello. All animal work was performed according to institutional guidelines and supervised by a veterinarian.

### Induction of Gestational Hpx in Mice

Six- to eight-week-old C57BL/6 mice were checked for vaginal plugs the day after mating. Mice with vaginal plugs were considered to be pregnant and that day was assigned as pregnancy day 1 (E1). From E10 to E15 mice were treated with 0.02% methimazole (MMI) (M8506, Sigma-Aldrich, USA) in the drinking water. In the control group, mice drank water without MMI during the entire pregnancy. A third experimental group consisted of pregnant mice that received MMI and T_4_ (2 mg/l) in the drinking water from E10 to E15. To analyze the health status of the offspring, a complete blood cells (CBC) analysis from blood samples of mice at 55 postnatal day was performed. Briefly, blood samples were obtained from the face vein of control, Hpx, and Hpx + T_4_ mice. Red blood cell count and white blood cell count were performed by an external veterinary laboratory (VetLab) and the obtained results were analyzed and resumed in Table S1 in Supplementary Material.

### Detection of THs and TSH

Thyroid hormones of mice and their respective progeny were measured on the last day of treatment (E15) and at postnatal day 55 (P55), respectively, from blood samples (500 µl) obtained from the tail. Serum tT_3_ and tT_4_ were measured by radioimmunoassay using Coat-A-Count Siemens Healthcare Diagnostics kits (cat no. TKT41 for tT_3_ and TKT31 for tT_4_). TSH was measured using a mouse ultrasensitive TSH enzyme-linked immunosorbent assay kit from Mybiosource (cat no. MBS704901), according to the manufacturer’s instructions.

### EAE Induction and Assessment

Seven-week-old female offspring gestated in Hpx, Hpx + T_4_, and euthyroidism (control) which weights were between 17 and 25 g were induced with a mild form of EAE. Briefly, these mice were s.c injected in the flank with 50 µg of myelin oligodendrocyte-glycoprotein-derived peptide [myelin oligodendrocyte glycoprotein (MOG)_35–55_, MEVGWYRSPFSRVVHLYRNGK] emulsified in complete Freund’s adjuvant supplemented with heat-inactivated *Mycobacterium tuberculosis* H37 RA. On this day and 48 h later, these mice also received an i.p. injection of 350 ng of Pertussis toxin. From day 1, the clinical score of all mice was derived according to standard reported score criteria (18). Mice were weighted before and each day after EAE induction. After 21 days of EAE induction mice were sacrificed for experimental analysis.

### Immunofluorescence for Myelin Basic Protein (MBP) and CD4^+^ and CD8^+^

At day 21st after EAE induction spinal cords from the three experimental groups were fixed in 4% PFA and frozen in OCT with isopentane in liquid nitrogen. Twenty-micron-thick lumbar sections were obtained using a cryostat (Leyca CM152S) and the tissue sections were fixed in 4% PFA. Primary antibody used for MBP assessment was MBP (3 µg/ml, Abcam, Cambridge, MA, USA) and samples were then incubated with Alexa594 antibody (10 µg/ml, Invitrogen). For cell infiltration analysis, primary antibodies were CD4-Alexa488 (5 µg/ml, Invitrogen), CD8 (5 µg/ml, Abcam, Cambridge, MA, USA), and nucleuses were counterstained with DAPI. Alexa647 antibody (10 µg/ml Invitrogen) was used as secondary antibody. Immunofluorescence was analyzed using a Fluoview FV1000 laser scanning confocal microscope (Olympus) with 20× objective. Briefly to calculate the demyelination score, at least 6 CNS tissue sections were analyzed and demyelination was scored according to Zappia et al. (19). 1 = traces of subpial demyelination, 2 = marked subpial and perivascular demyelination, 3 = confluent perivascular or subpial demyelination, 4 = massive perivascular and subpial demyelination involving one half of the spinal cord with presence of cellular infiltrate in the CNS parenchyma, and 5 = extensive perivascular and subpial demyelination involving the whole cord section with presence of cellular infiltrate in the CNS parenchyma.

CD4 or CD8 positive T cells were quantified in a blind fashion from three independent experiments, where three different areas were chosen from three lumbar sections per mice. Positive CD4 or CD8 cells were expressed as the percentage from the total cells stained with DAPI in each section.

### MOG-Specific T Cell Cytokine Release Assays

Splenocytes obtained on day 21 after EAE induction were cultured (2.0 × 10^5^ cells/well) in RPMI 1640 medium containing 5% FBS with MOG_35–55_ (10 µM). Cultures were incubated in 96-well flat bottom plate for 48 h, 72 h, and 5 days at 37°C in a cell culture incubator. IL-10 release in response to MOG_35–55_ was determined on culture supernatants by ELISA following manufacturer’s instructions (eBioscience).

### Quantification of CD4^+^CD25^+^FOXP3^+^ and IL-10 by Flow Cytometry

Total splenocytes were stained with anti-CD4-PE-Cy7 (clone RM4-5; Biolegend, San Diego, CA, USA), anti-CD25-PE (clone PC61.5; eBioscience Inc., San Diego, CA, USA), and fixed in 1% PFA in PBS. Then, cells were incubated with anti-Foxp3-APC (clone FJK-16s; eBioscience Inc., San Diego, CA, USA) and anti-IL-10-FITC (BD Bioscience 554466) in saponine 0.1% and BSA 3% and fixed again in PFA 1%.

Cells from the immune system were isolated from brain and spinal cord from the three experimental groups. Before the extraction of these organs, mice were intracardially perfused with PBS. Then, the meninges were discarded, and the brain and spinal cord were homogenized in a PBS-EDTA 2 mM solution by passing through a 70 μm cell strainer (Bioscience Inc., San Diego, CA, USA). The samples were centrifuged and the pellet was re-suspended in a 30% Percoll solution and loaded onto a 70% solution without mixing the phases. The samples were centrifuged again at 700 × *g* for 20 min at RT and the interface was recovered and centrifuged to pellet the immune cells that were re-suspended in a PBS-5% FBS solution.

Cells of the immune system were isolated from blood samples by adding ACK to each sample for 5 min at RT. The samples were centrifuged at 500 × *g* for 5 min and the pellet was re-suspended again in ACK for 5 min at RT. To neutralize ACK, PBS was added to the samples and they were centrifuged at 500 × *g* for 5 min and the pellet was re-suspended in PBS-5% FBS. Cells were acquired using a FACSCanto II (BD Bioscience) and data were analyzed using FCS Express 4 research edition software (*De Novo*).

#### Content of IL-10 in the Blood, Spleen, and CNS

The content of IL-10 was determined by ELISA in blood, spleen, and CNS. First the protein concentration was determined in blood, spleen, and CNS using the BCA method. IL-10 concentration was determinated by ELISA (IL-10 ELISA Set BD OptEIA 6154834) following the manufacturer’s instructions. 500 µg of total protein was used for IL-10 quantification. To determine the IL-10 level in the serum, blood samples were obtained from the vena cava from mice. The blood was left for 30 min at 4°C to clot and then it was centrifuge at 10,000 × *g* at 4°C. A 100 µl of serum were used to measure IL-10.

#### *In Vitro* Differentiation of Naïve CD4^+^ T Cells to CD4^+^CD25^+^FOXP3^+^ T Cells

To induce the differentiation of T_reg_ cells a single-cell suspension of spleens of Hpx, Hpx + T_4_, and control mice was obtained using ACK. CD4^+^CD25^−^ T cells were enriched using the CD4^+^CD25^+^ regulatory T cell Isolation Kit (MACS^®^ 130-091-041) following the manufacturer’s instruction to obtain enriched CD4^+^CD25^−^ T cell solution. The single-cell suspension was cultured in triplicates in induction (i) T_reg_ medium with activation factors αCD3 and αCD28 for 5 days at 37°C and 5% CO_2_. To further analyze the differentiation capacity of T_reg_ cells, CD4^+^CD25^−^ T cells were also cultured in iT_reg_ medium without activation factors and in cRPMI 1640 medium only as control conditions. Complete RPMI 1640 medium serves as basis of all media. For the iT_reg_ medium IL-2 (20 ng/ml, BD Biosciences 550069) and mTGF-β1 (5 ng/ml) and for T_reg_ cell activation αCD3 (2 µg/ml) and αCD28 (2 µg/ml) were added. To compare the absolute number of CD4^+^CD25^+^IL-10^+^FoxP3^+^ T cells that had differentiated in the culture, the absolute number of CD4^+^CD25^+^IL-10^+^FoxP3^+^ T cells found in the culture with cRPMI 1640 medium was subtracted from the absolute number of CD4^+^CD25^+^IL-10^+^FoxP3^+^ T cells found in the culture with the T_reg_ induction media. The amount of IL-10 secreted in the *in vitro* T_reg_ generation assay was determined by ELISA. For that 100 µl of supernatant of each sample was used in duplicate to measure IL-10 by ELISA following the manufacturer’s instructions as previously described. To compare the amount of IL-10 being produced during this *in vitro* T_reg_ generation assay the amount of IL-10 found in the culture of CD4^+^CD25^−^ T cells with cRPMI 1640 medium was subtracted from the amount of IL-10 detected in the culture of CD4^+^CD25^−^ T cells with the T_reg_ induction media.

### Purification of CD4^+^CD25^+^ T Cells for Suppression Assays and Adoptive Transfer Experiments

CD4^+^CD25^+^ T cells were isolated from spleen as previously described (20). Briefly, CD4^+^ T cells were isolated by negative selection from cell suspensions of spleen by using magnetic-associated cell sorting beads (MACS, Mylteni Biotec, Germany no. catalog 130-091-041). Then, from the pool of CD4^+^ T cells, CD25^+^ T cells were purified by positive selection using magnetic associated cell sorting beads (MACS, Mylteni Biotec, Germany no. catalog 130-091-041) and separated from CD25^−^ T cells, which correspond to T effector cells (T_Eff_ cells). The purification efficiency of CD4^+^CD25^+^ or CD4^+^CD25^−^ T cells was analyzed by flow cytometry by using 0.08 µg/ml of anti-CD4^+^ monoclonal antibody coupled to allophycocyanin (BD Pharmamingen, no. catalog 553051) and 0.08 µg/ml anti-CD25^+^ monoclonal antibody coupled to phycoerythrin (eBioscience no. catalog 12-0232).

### *In Vitro* Suppression Assays

CD4^+^CD25^+^ T cells isolated from spleen as previously described were used to measure their suppression capacity over CD4^+^CD25^−^ T cells (T_Eff_) proliferation. For suppression assays, CD4^+^CD25^+^ T cells were incubated with T_Eff_ cells previously stained with 5 µM of carboxyfluorescein diacetate succinimidyl ester (CFSE) (Molecular Probes, n° catalog C34554) per 10 min at 37°C in RPMI 1640 medium. The suppression assay was performed in a 96-well coated plate by co-culturing 50,000 T_Eff_ cells stained with CFSE with 5,000, 50,000, or 100,000 of T_reg_ cells for 72 h at 37°C in a humidified atmosphere with 5% CO_2_. This co-culture was performed in the presence of RPMI 1640 medium supplemented with 5% heat-inactivated fetal bovine serum, 100 IU/mL penicillin–streptomycin, 2 mM l-glutamine, 1 mM Hepes, 1 mM sodium pyruvate, 1 mM non-essential amino acids, 55 µM 2-mercaptoethanol (all from Gibco), 0.25 µg/ml of anti-mouse CD3, and 0.25 µg/ml of anti-mouse CD28 (BD Pharmamingen catalog 553058/553295) to activate T_Eff_ cells. 50 IU of IL-2 (eBioscience, no. catalog 14-8021) was also added to the medium to keep T_reg_ cells properly. After 72 h of incubation, the cells were centrifuged at 300 *g* per 5 min, then the cells were re-suspended in PBS with 5% FBS. The CFSE fluorescence intensity was analyzed in BD Canto II flow cytometer (Pontificia Universidad Católica de Chile, Departamento de Ciencias Biológicas). The data obtained were analyzed using Flowjo Software (Flowjo 7.6.1). Singlets were selected from total FSC/SSC events (10,000 events). The method of division index was used to calculate the percentage of suppression (21). Briefly, the percentage of suppression capacity is calculated as follows: from the Flowjo software values of division indices (DI) are used in the formula. DI for T_Eff_ cells in the presence of T_reg_ cells (DI T_Eff_ in the presence of T_reg_) and DI for T_Eff_ cells alone (DI of T_Eff_ alone):

$$\%\text{ Suppression}=100-\left(\frac{{\text{DIT}}_{\text{Eff}}\text{ in the presence of Treg}}{{\text{DIT}}_{\text{Eff}}\text{ alone}}\right)\times 100.$$

### Adoptive Transfer of T_reg_ Cells

For the adoptive transfer experiments, first EAE was induced to control and Hpx offspring as previously described. At day 21 of EAE the spleens were isolated and single-cell suspensions of splenocytes were prepared as described before. Then CD4^+^CD25^+^ T or T_reg_ cells were isolated from mouse splenocytes with a CD4^+^CD25^+^ regulatory T cell Isolation kit (Miltenyi Biotec) as previously described. Then 6 × 10^5^ cells per well of CD4^+^CD25^+^ T cells were stimulated by culture with 2,000 IU/ml recombinant mouse IL-2 (R&D Systems) for 7 days in 24-well plate which were pre-coated with 1 µg/ml of anti-mouse CD3 and anti-mouse CD28 Abs (BD Pharmingen). The phenotype of T_reg_ cells was confirmed by flow cytometry using CD4^+^ and Foxp3 markers before adoptive transfer into mice (Figure 7A). 1 × 10^6^ cells positive for CD4^+^CD25^+^FOXP3^+^ were intravenously injected to recipient mice gestated in Hpx or control. 24 h later EAE was induced in these recipient mice as previously described. Clinical scores for EAE assessment was monitored for 21 days in the recipient mice as previously described.

### Statistical Analyses

Data and statistical analyses were performed using Prism 4 software (GraphPad Software, Inc.) and Statistica 6.0 software (StatSoft Inc., 2001, Tulsa, OK, USA). The results are shown as mean ± SEM. Statistical differences for THs and TSH were tested using unpaired Student’s *t*-test. Repeated measure ANOVA test was used to analyze the effect of gestational Hpx on EAE clinical scores. Statistical significance is indicated by *p < 0.05, **p < 0.01, and ***p < 0.001.

All data were tested for normality and homoscedasticity using Kolmogorov–Smirnov and Levene’s tests. When necessary, data were transformed to meet statistical assumptions. When differences were significant at *P* < 0.05 after general linear model tests, we used *a posteriori* Bonferroni test for multiple comparisons and Tukey’s test for balanced samples.

## Results

### MMI Treatment Induces Hpx in Pregnant Mice

**Figure 1:**
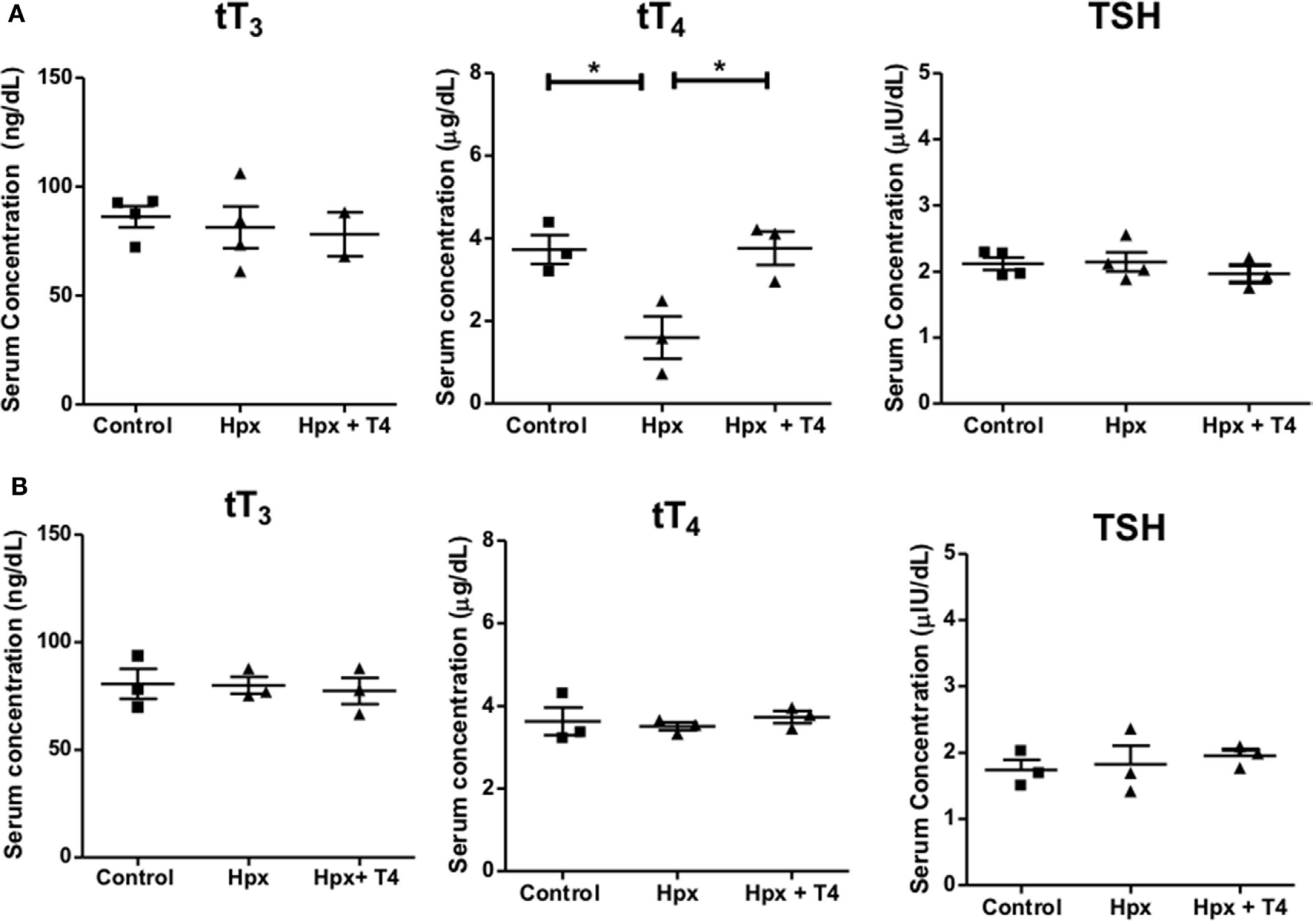
Treatment with methimazole induces hypothyroxinemia (Hpx) in pregnant mice. **(A)** tT_4_, tT_3_, and thyroid stimulating hormone (TSH) levels were analyzed from serum samples of pregnant mice at E15 after treatment with methimazole (MMI). The treated group with MMI was designated as Hpx. The group treated with MMI plus T_4_ was named Hpx + T_4_, and the untreated pregnant mice as control group. Control *N* = 4, Hpx *N* = 4, and Hpx + T_4_ *N* = 3. **(B)** tT_4_, tT_3_, and TSH levels were analyzed from serum samples of the offspring from the three experimental groups at P55: (1) gestated in Hpx; gestated in euthyroidism (control); or gestated in MMI plus T_4_ (Hpx + T_4_) conditions. Data are showed as the mean ± SEM. *p < 0.01. Control *N* = 3, Hpx *N* = 3, and Hpx + T_4_ *N* = 3.

Gestational Hpx was induced by the administration of MMI to pregnant mice (see Materials and Methods). To demonstrate that pregnant mice suffered Hpx during gestation, serum tT_3_, tT_4_ and TSH were measured as described in Section “Materials and Methods.” The mean serum levels for tT_3_ and TSH remained unchanged between pregnant mice treated with MMI in comparison to pregnant mice without treatment (control) or those that received MMI and T_4_ (Hpx + T_4_) (Figure 1A). On the other hand, the mean serum levels of tT_4_ in the pregnant mice treated with MMI were reduced compared to the mean values of Hpx + T_4_ and control mice (Figure 1A). These results indicate that the treatment with MMI to pregnant mice induced Hpx a condition that was reverted by the addition of T_4_ during the treatment with MMI. The thyroidal state was also analyzed in the offspring at adult age of P55. The serum levels of tT_3_, tT_4_, and TSH remained similar in the three offspring experimental groups (Figure 1B) indicating that the MMI treatment during gestation does not affect the thyroid function of progeny at the adulthood.

### Female Offspring Gestated in Hpx Show More Intense EAE Symptoms

**Figure 2:**
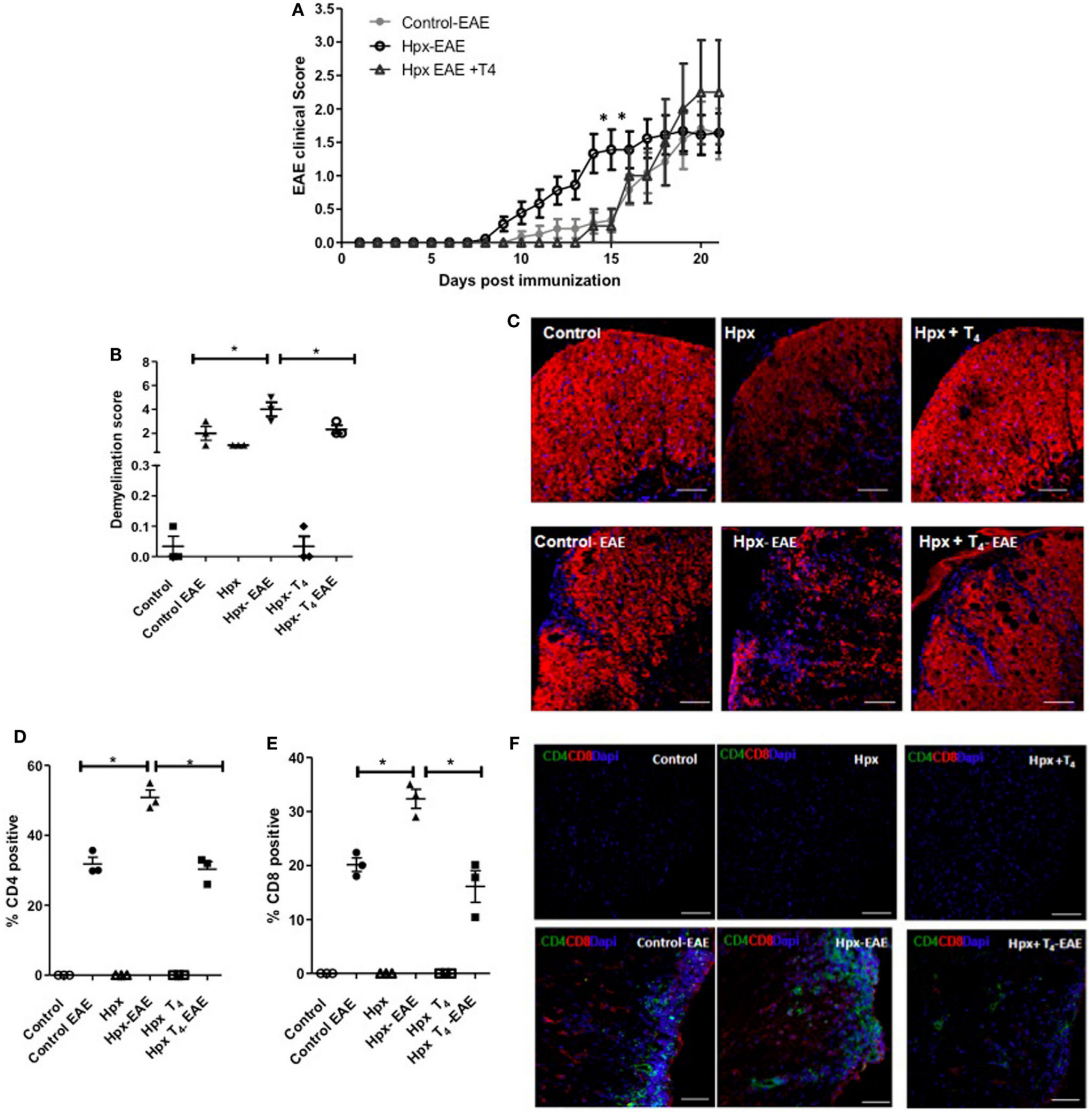
The offspring gestated in hypothyroxinemia (Hpx) has an intense experimental autoimmune encephalomyelitis (EAE). **(A)** EAE was induced in C57BL/6 female offspring that were gestated under Hpx (Hpx empty circles, *N* = 18), or gestated under euthyroidism (control, filled circles, *N* = 12), or gestated under treatment with MMI + T_4_ (Hpx + T_4_, empty triangles, *N* = 8). The severity of EAE was plotted base on a daily pathological score (see Materials and Methods) every day post-EAE induction. The mean of the disease scores for the female offspring and ± SD was plotted. Statistical analysis was performed by two-way ANOVA, post-test Bonferroni. *p < 0.05. **(B)** Demyelination score from quantitative histopathological analyses of spinal lumbar cord sections stained for myelin basic protein (MBP) from mice at 21 days after EAE induction. Values represent the mean ± SEM, *N* = 3, *p < 0.05. **(C)** Representative confocal microscopy pictures of white matter from spinal lumbar cord of control, Hpx, or Hpx + T_4_ offspring that suffer or not of EAE were analyzed for MBP (red). Nuclei are shown in blue (DAPI). Bar size is 100 µm. **(D)** Quantitative analysis of CD4^+^ and **(E)** CD8^+^ T cells detected by confocal immunofluorescence of spinal cord lumbar sections of control, Hpx, and Hpx + T_4_ offspring after 21 days of induction with EAE. Values represent the mean ± SEM. *p < 0.05 (*N* = 3). **(F)** Representative confocal microscopy images of lateral lumbar sections of the spinal cord regions immunostained with anti-CD4 (green), anti-CD8 (red), and DAPI (blue) of control, Hpx, and Hpx + T_4_ offspring that were induced or not with EAE. The sections were analyzed with 20× objective. Bar size is 100 µm (*N* = 3).

Experimental autoimmune encephalomyelitis was induced in the progenies of Hpx, Hpx + T_4_, and control at P55 (see Materials and Methods) and the clinical score was followed for 21 days. As shown in Figure 2A, both control and Hpx + T_4_ groups displayed a similar pattern both for the onset and the intensity of EAE symptoms. In contrast, the Hpx group showed an earlier onset of the disease at day 8 and the clinical score was significantly higher at days 14–15 when compared to Hpx + T_4_ and control groups. Nevertheless, all the experimental groups reached a similar clinical score between 2.0 and 2.5 by the final days of evaluation (Figure 2A). Spinal cords from the offspring of the three experimental groups at day 21 after EAE induction were stained for MBP to evaluate demyelination (Figures 2B,C). Fluorescence quantification analyses showed that the progeny gestated in Hpx conditions has increased the mean demyelination score compared to the offspring gestated in euthyroidism or Hpx + T_4_ condition (Figures 2B,C). The demyelination score increased significantly in the offspring of the three experimental groups when EAE was induced (Figures 2B,C). However, the mean score for Hpx gestated mice increases to 4, while the increase observed in control and Hpx + T_4_ gestated mice reaches a mean score of 2, which is a statistically significant decrease (Figure 2B). Cellular infiltration in spinal cord sections were analyzed at day 21 after EAE induction by confocal microcopy for anti-CD4, anti-CD8, and anti-DAPI antibodies (Figures 2D–F). Quantification analyses showed CD4^+^ and CD8^+^ T cells only in the offspring from the three experimental groups that suffer EAE (Figures 2D–F). A significant increase in the percentage of both CD4^+^ and CD8^+^ T cells was detected at the spinal cords from the offspring gestated in Hpx that suffer EAE compared to the offspring gestated in control or Hpx + T_4_ that suffer EAE (Figures 2D–F). To rule out the possibility that gestational Hpx induced an impaired physical condition or unhealthy state in their offspring that could affect the outcome of EAE, the weight of all the experimental groups was registered before and through the EAE experiment and a cell blood count (CBC) test was performed at postnatal day 55. Figure S1 in Supplementary Material showed that the offspring gestated in Hpx has similar weight compared to the offspring gestated in Hpx + T4 and euthyroidism at P55. The Figure S2 in Supplementary Material showed that weight of the three experimental groups during EAE was similar. Moreover, the offspring gestated in Hpx showed similar CBC compared to the offspring gestated in Hpx + T4 and euthyroidism (See Table S1 in Supplementary Material). These results support the notion that the offspring gestated in Hpx showed a more intense EAE than the offspring gestated in euthyroidism and that it is not due to an unhealthy state.

### The Spleen of Offspring Gestated in Hpx Has Similar Percentage of CD4^+^CD25^+^FOXP3^+^ and Low Content of IL-10 After EAE Induction

**Figure 3:**
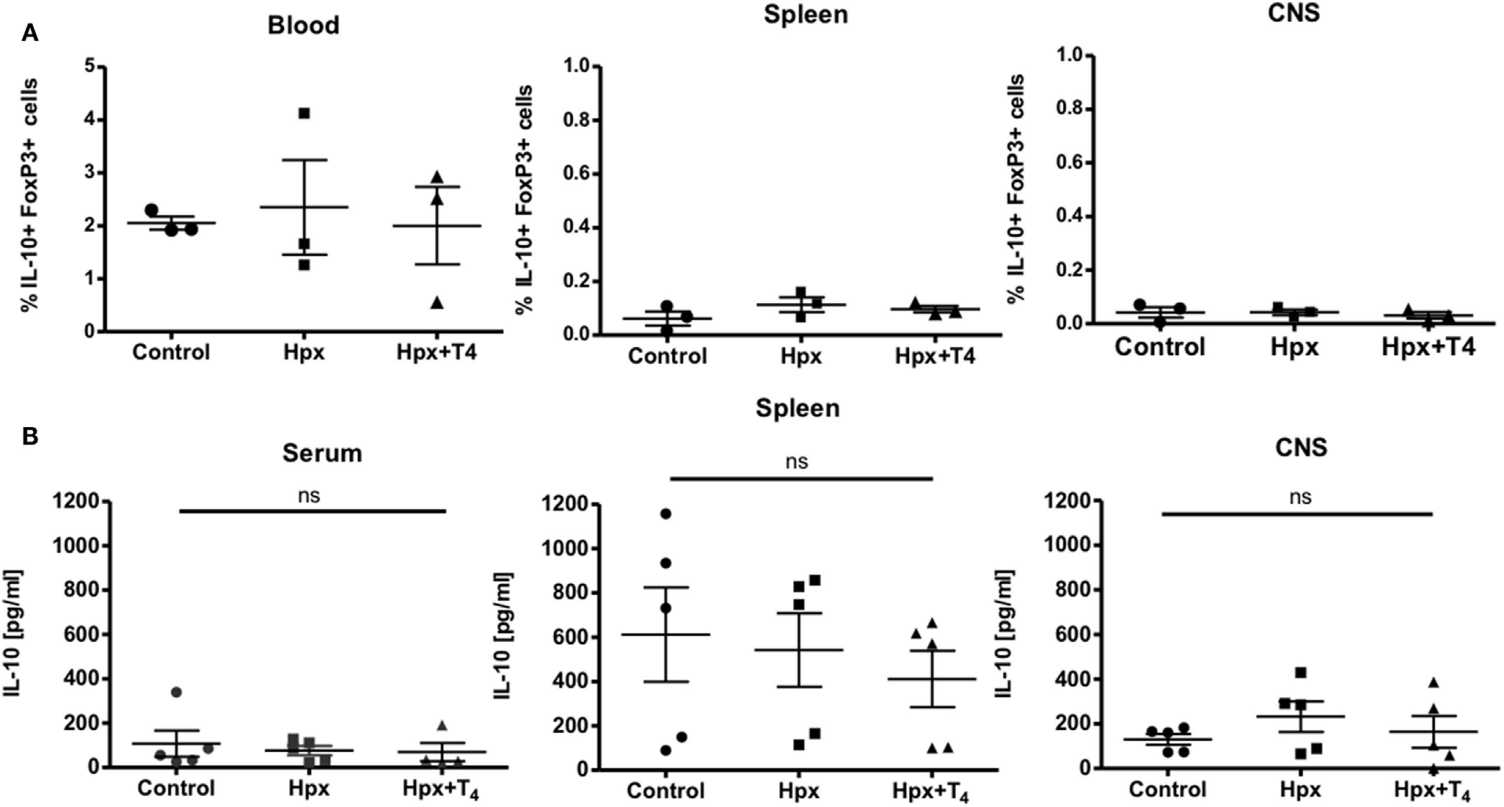
The percentage of CD4^+^CD25^+^FOXP3^+^IL-10^+^ and the content of IL-10 in the blood, spleen, and central nervous system (CNS) is similar in the offspring gestated in hypothyroxinemia (Hpx) or euthyroidism. **(A)** Determination of frequency of CD4^+^CD25^+^IL-10^+^FoxP3^+^cells in blood, spleen, and CNS of control, Hpx, and Hpx + T_4_ mice. Statistical analysis showed no significant differences in frequency of CD4^+^CD25^+^IL-10^+^FoxP3^+^cells of Hpx mice compared to control and Hpx + T_4_ mice. Control and Hpx *n* = 7, Hpx + T_4_ *n* = 3. Mean ± SEM *p < 0.05. ANOVA and Tukey’s test. **(B)** Determination of the content of IL-10 in the serum, spleen, and CNS of the offspring gestated in control, Hpx, and Hpx + T_4_. Statistical analysis showed no significant differences in the content of IL-10 of Hpx gestated in Hpx compared to control and Hpx + T_4_ mice. *n* = 3, mean ± SEM *p < 0.05, ANOVA, and Tukey’s test.

**Figure 4:**
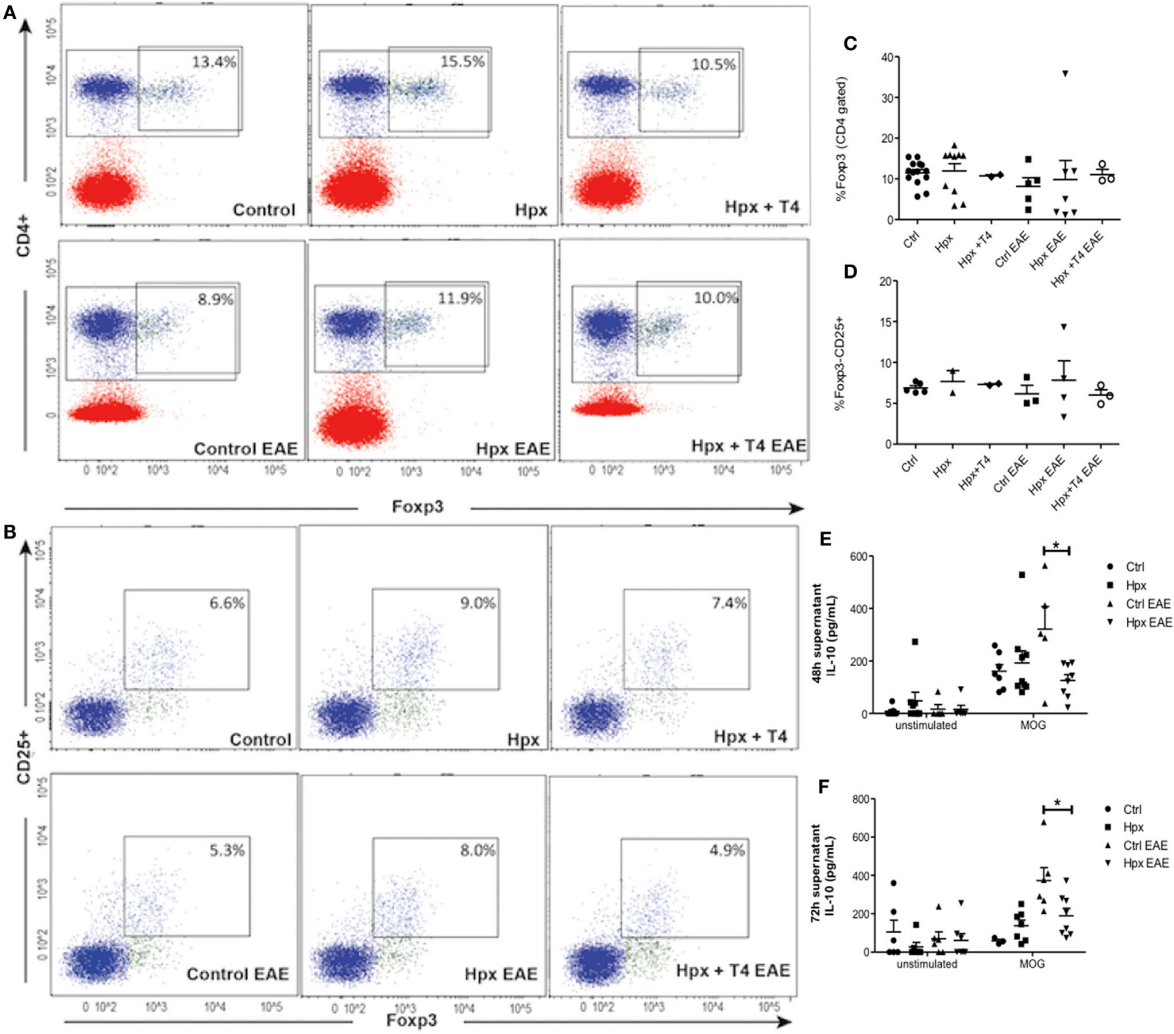
The spleen of the offspring gestated in hypothyroxinemia (Hpx) has similar percentage of CD4^+^CD25^+^FOXP3^+^ T cells in the spleen of the offspring gestated in Hpx suffering or not experimental autoimmune encephalomyelitis (EAE) and less content of IL-10 only after EAE induction. **(A)** Representative dot plots of CD4^+^Foxp3^+^ T cells from spleens of the three experimental groups gestated in euthyroidism (control), Hpx, and Hpx + T_4_ induced or not with EAE. This study was performed after 21 days of EAE induction. The upper right panel shows the percentage of CD4^+^Foxp3^+^ T cells from spleens in each experimental group. **(B)** Representative dot plots of CD4^+^CD25^+^Foxp3^+^ T cells from spleens of the three experimental groups gestated in euthyroidism (control), Hpx, and Hpx + T_4_ induced or not with EAE. The upper right panel of every dot-plot shows the percentage of CD4^+^Foxp3^+^CD25^+^ T cells. The graphs **(C,D)** show the analysis for the dot plots **(A,B)**, respectively. These graphs correspond to three independent experiments that used five mice per experimental group. **(E,F)** IL-10 secretion measured by ELISA from the supernatants of total splenocytes after being cultured for 48 or 72 h, respectively either in the absence or presence of myelin oligodendrocyte glycoprotein. The splenocytes are derived from the three experimental groups gestated in euthyroidism (control), Hpx, or Hpx + T_4_ induced or not with EAE. Values represent the mean ± SEM. Statistical analysis was performed using one-way ANOVA and Tukeys post-test. Statistical significance is indicated as *p < 0.05 (*n* = 3).

Given that it has been reported that the number and function of T_reg_ cells can affect the autoimmune response (22–24), these factors were analyzed in the offspring gestated in Hpx. First, the percentage of CD4^+^CD25^+^FOXP3^+^IL-10^+^ T cells was analyzed in the blood, spleen, and CNS (Figure 3A). The offspring gestated in Hpx showed similar percentage of CD4^+^CD25^+^FOXP3^+^IL-10^+^ T cells in these three organs compared to control and Hpx + T4 (Figure 3A). The content of IL-10 analyzed in blood, spleen, and CNS of the offspring gestated in Hpx showed similar levels compared to the offspring gestated in control or Hpx + T4 (Figure 3B). To analyze whether the induction of EAE could change this T cell population, the percentage of CD4^+^FOXP3^+^ T cells and the percentage of CD25^+^FOXP3^+^ T cells in the spleen of the offspring from the three experimental groups was analyzed by flow cytometry after 21 days of EAE induction (Figures 4A,B). The percentage of CD4^+^Foxp3^+^ T cells was similar in the offspring of the three experimental groups (Figure 4C). Similarly, the percentage of CD4^+^Foxp3^+^CD25^+^ cells from spleens was similar for the offspring of the three experimental groups (Figure 4D). The secretion of IL-10 was analyzed from the supernatants of splenocytes primary cell culture. These splenocytes were derived from the offspring of the three experimental groups that suffered or not EAE. The culture of splenocytes was treated with MOG_35–55_ to induce T cell activation (25). High levels of IL-10 secretion were observed in those supernatants that the splenocytes were stimulated with MOG_35–55_ (Figures 4E,F). However, the splenocytes derived from the offspring gestated in Hpx that suffered EAE had significantly lower IL-10 secretion as compared to mice gestated in euthyroidism suffering EAE (Figures 4E,F). These results suggest that the percentage of T_reg_ cells from the spleen is not altered in the offspring gestated in Hpx whether they suffer or not EAE. Moreover, the low levels of IL-10 in the spleen of offspring gestated in Hpx that suffer EAE could suggest that the suppressive capacity is impaired in these mice.

### CD4^+^CD25^+^ T Cells From the Spleen of Their Offspring Gestated in Hpx Have Reduced Capacity to Suppress Activated T_Eff_ Cells

**Figure 5:**
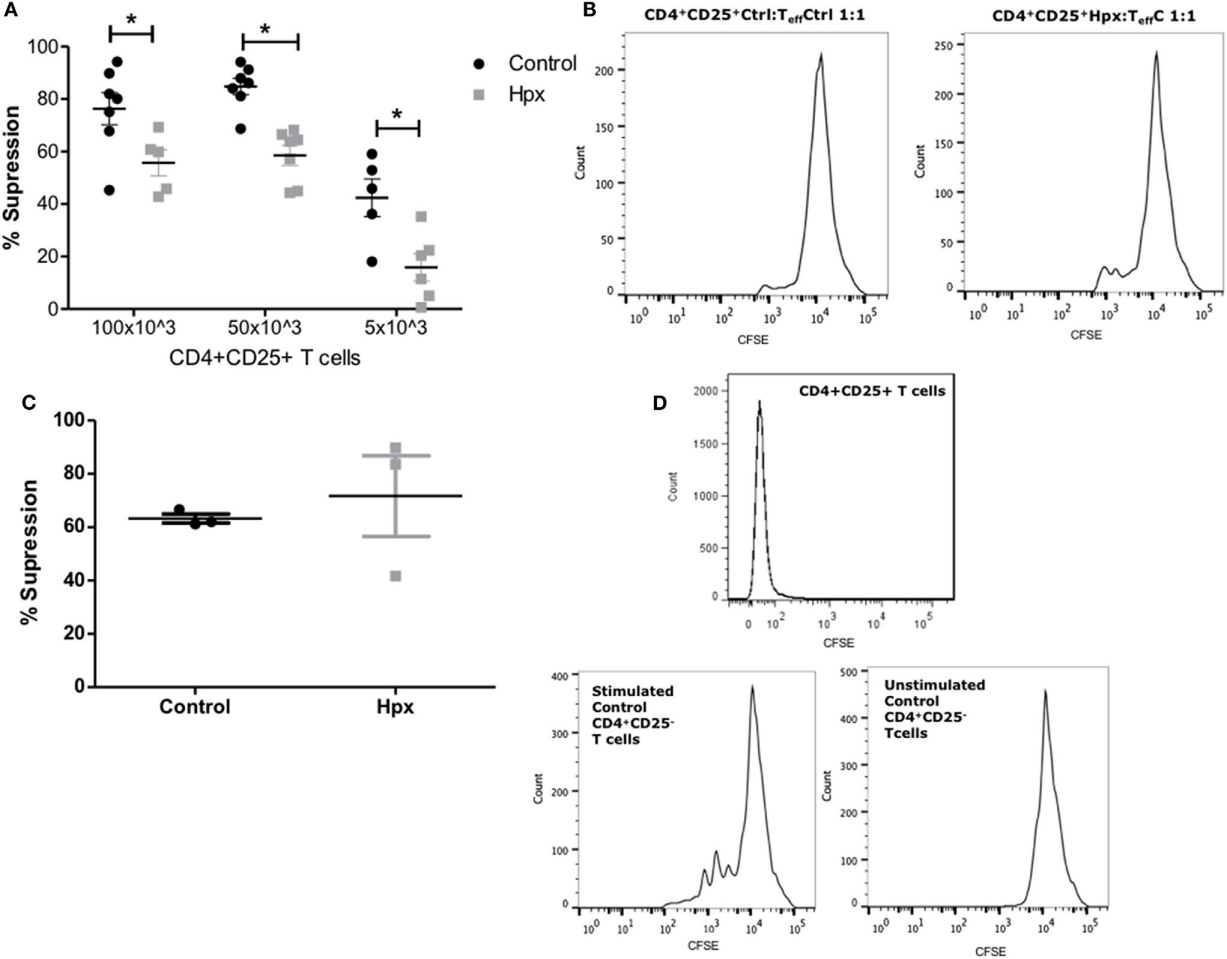
CD4^+^CD25^+^ T cells from the spleen of their offspring gestated in hypothyroxinemia (Hpx) have reduced capacity to suppress activated effector T cells (T_Eff_) cells. **(A)** The graph shows the percentage of suppression capacity of CD4^+^CD25^+^ T cells from spleen of the offspring gestated in euthyroidism (control) or Hpx over T_Eff_ cells stained with carboxyfluorescein diacetate succinimidyl ester (CFSE) from control mice and activated with anti-CD3 and anti-CD28 monoclonal antibodies *N* = 6 independent cultures (see Materials and Methods). **(B)** The right histogram is representative of T_Eff_ cells from control mice loaded with CFSE to analyze proliferation in the presence of CD4^+^CD25^+^ T cells from control offspring (left); the right histogram is representative of T_Eff_ cells from control mice loaded with CFSE to analyze proliferation in the presence of CD4^+^CD25^+^ T cells derived from Hpx offspring. **(C)** The graph shows the percentage of suppression capacity of CD4^+^CD25^+^ T cells from spleen of the offspring gestated in euthyroidism (control) over CD4^+^CD25^+^ T cells stained with CFSE from control or Hpx offspring that were activated with anti-CD3 and anti-CD28 monoclonal antibodies *N* = 3 independent cultures (see Materials and Methods). Values represent the mean ± SEM. Statistical analysis was performed using one-way ANOVA and Tukeys post-test. Statistical significance is indicated as *p < 0.05. **(D)** Representative histogram of the autofluorescence for CD4^+^CD25^+^ T cells from control offspring mice (upper); representative histogram of T_Eff_ cells from control mice that were stimulated with anti-CD3 and anti-CD8 antibodies and without CD4^+^CD25^+^ T cells (middle); and representative histogram of T_Eff_ cells without stimulation with anti-CD3 and anti-CD28 antibodies and without CD4^+^CD25^+^ T cells (left).

The suppression of T_Eff_ cells proliferation was measured in the presence of CD4^+^CD25^+^ T cells derived from the spleen of mice gestated in Hpx in an *in vitro* assay as described in Section “Materials and Methods.” The CD4^+^ CD25^+^ T cells derived from the spleen of offspring gestated in control or Hpx were isolated and co-cultured with T_Eff_ cells from control mice. The T_Eff_ cells (CD4^+^ CD25^−^ cells) were previously labeled with CFSE (see Materials and Methods) in the co-culture were activated with the presence of soluble anti-CD3 and anti-CD28 antibodies. The percentage of suppression of T_Eff_ cells proliferation in the presence of CD4^+^CD25^+^ T cells was plotted in the Figure 5A. The suppression of T_Eff_ cells proliferation was significantly reduced in the presence of CD4^+^CD25^+^ T cells derived from the offspring gestated in Hpx compared to the offspring gestated in euthyroidism (control) at different ratios (Figure 5A). Representative histograms are shown in Figure 5B. The left and right histograms represent the proliferations of T_Eff_ cells from control mice in the presence of CD4^+^CD25^+^ T cells from control or Hpx offspring, respectively (Figure 5B). The highest peaks of both figures represent the number of T_Eff_ cells that had not proliferated. The small peaks at 10^3^ of intensity represent the T_Eff_ cells that proliferated. The T_Eff_ cells that are co-cultured with CD4^+^CD25^+^ T cells from the offspring gestated in Hpx had a high number of cells that proliferate (right histogram) in comparison to those co-cultured with CD4^+^CD25^+^ T cells from control offspring (left histogram). Aiming to elucidate the proliferation capacity of T_Eff_ cells from the offspring gestated in Hpx, T_Eff_ cells from these mice, or from the offspring gestated in control were co-cultured in the presence of CD4^+^CD25^+^ T cells from control mice (Figure 5C). The analysis of these results showed that T_Eff_ cells from the offspring gestated in Hpx have similar capacity to proliferate than T_Eff_ cells from control offspring in the presence of CD4^+^CD25^+^ T cells from control mice (Figure 5C). Thus, the analysis of these results suggests that gestation in Hpx would reduce the suppressive capacity of CD4^+^CD25^+^ T cells from its offspring. However, gestational Hpx will not alter the capacity of T_Eff_ cells of its offspring to proliferate. Figure 5D shows representative histograms for: (1) the peak of CD4^+^CD25^+^ T cells from control offspring, that corresponds to its autofluorescence (upper); (2) the proliferation of T_Eff_ cells from control mice cultured with anti-CD3 and anti-CD8 antibodies and without the presence of CD4^+^CD25^+^ T cells (left); and (3) the proliferation of T_Eff_ cells from control mice cultured without anti-CD3 and anti-CD28 antibodies and without CD4^+^CD25^+^ T cells (right).

### CD4^+^CD25^−^ T Cells From the Offspring Gestated in Hpx Show a Reduced Capacity to Differentiate Toward CD4^+^CD25^+^IL-10^+^FoxP3^+^ T Cells and to Secrete IL-10

**Figure 6:**
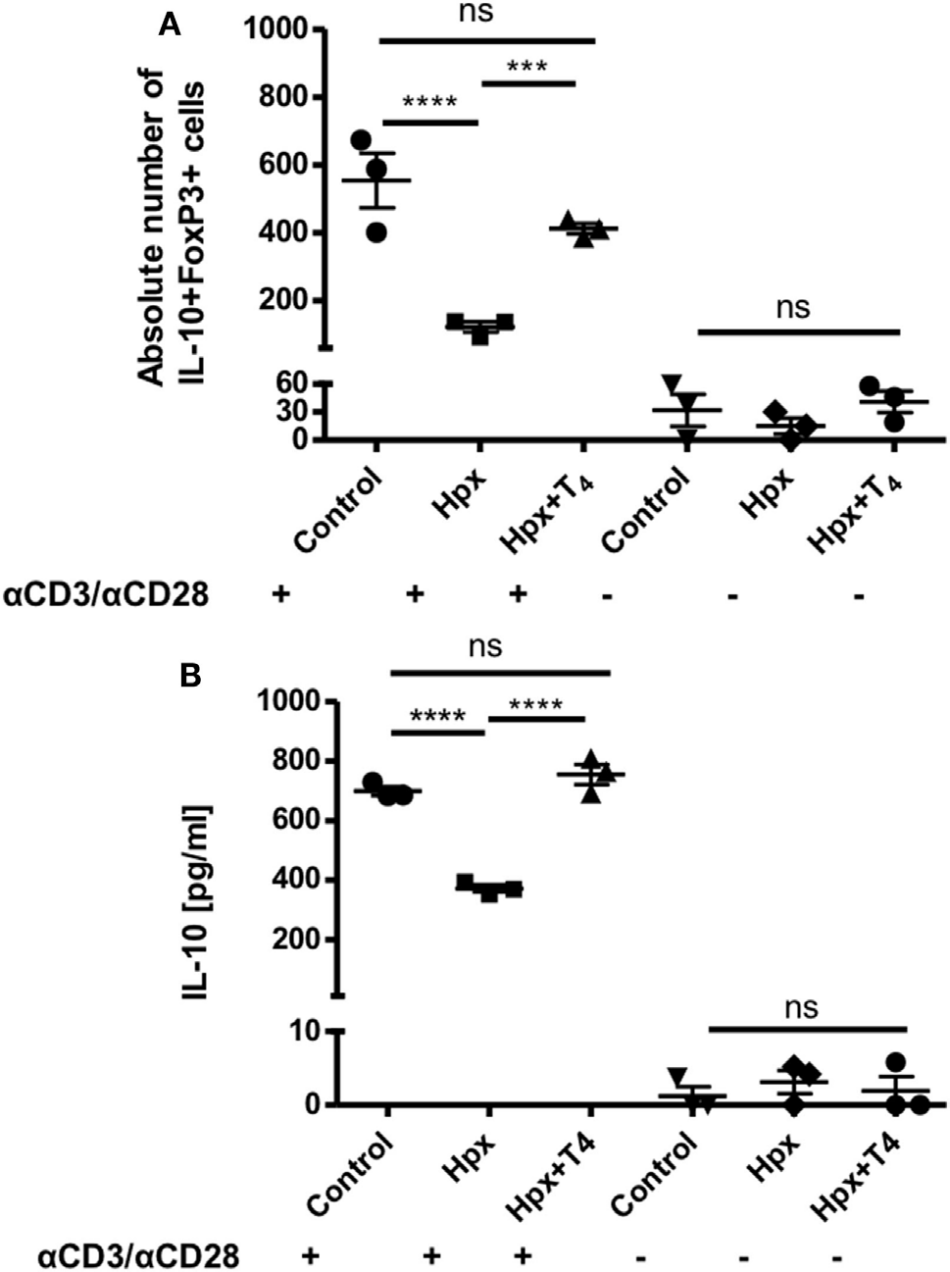
CD4^+^CD25^−^ T cells from mice gestated in HTX show a decreased differentiation capacity toward CD4^+^CD25^+^FOXP3^+^IL-10^+^ T cells *in vitro* assay. **(A)** The graph shows the absolute number of CD4^+^CD25^+^IL-10^+^FoxP3^+^ after an *in vitro* cell culture assay for regulatory T cell (T_reg_) generation. CD4^+^CD25^−^ T cells derived from the spleen of offspring gestated under control, Hpx, or Hpx + T_4_ were cultured *in vitro* for 5 days under induction media to stimulate the differentiation of T_reg_ in the presence or in the absence of anti-CD3 and anti-CD28 antibodies. Statistical analysis shows a significantly decrease in the absolute number of CD4^+^CD25^+^IL-10^+^FoxP3^+^ in the *in vitro* T_reg_ differentiation assay of CD4^+^CD25^−^ T cells derived from the offspring gestated in Hpx cells. There are not significant differences observed when CD4^+^CD25^−^ T cells were cultured in the induction medium for T_reg_ but without anti-CD3 and anti-CD28 antibodies *n* = 3, mean ± SEM *p* ≤ 0.05 ANOVA and Tukey’s test. **(B)** The graph shows the content of IL-10 measured by ELISA in the supernatant of CD4^+^CD25^−^ T cells from spleen of control, Hpx, and Hpx + T_4_ offspring. CD4^+^CD25^−^ T cells were *in vitro* cell cultured for 5 days under induction medium for T_reg_ and stimulated or not with anti-CD3 and anti-CD28 antibodies (see Materials and Methods). Statistical analysis shows a significantly reduced amount of IL-10 of cultured CD4^+^CD25^−^ T cells of Hpx mice compared to control and Hpx + T_4_ offspring. There are no significant differences observed when CD4^+^CD25^−^ T cells were cultured in the induction media for T_reg_ without anti-CD3 and anti-CD28 monoclonal antibodies. *n* = 3, mean ± SEM ***p < 0.001 and ****p < 0.0001, ANOVA, and Tukey’s test.

To analyze the differentiation capacity of CD4^+^CD25^−^ T cells toward CD4^+^CD25^+^IL-10^+^FoxP3^+^ T cells, CD4^+^ T cells were isolated from spleens of CTRL, Hpx, and Hpx + T_4_ mice. They were then cultured in medium for T_reg_ induction with and without activation factors (anti-CD3 and anti-CD28 antibodies, see Materials and Methods). Statistical analysis showed that CD4^+^CD25^−^ T cells derived from the offspring gestated in Hpx and cultured *in vitro* with medium for T_reg_ induction and anti-CD3 and anti-CD28 antibodies had significantly reduced the generation of CD4^+^CD25^+^IL-10^+^FoxP3^+^ T cells (Figure 6A). There was no significant difference observed in the absolute number of CD4^+^CD25^+^IL-10^+^FoxP3^+^ T cells with medium for T_reg_ induction without anti-CD3 and anti-CD28 antibodies in CTRL, Hpx, and Hpx + T_4_ mice (Figure 6A). The amount of IL-10 secreted in *in vitro* generation of T_reg_ assay from CD4^+^CD25^−^ T cells cultured in medium for T_reg_ induction was measured by ELISA (Figure 6B). Statistical analysis showed a significantly reduced amount of IL-10 in the cell culture supernatant of CD4^+^CD25^−^ T cells of Hpx mice cultured in iT_reg_ with activation factors (Figure 6B). There was no significant difference observed in the amount of IL-10 found in the cell culture supernatant of CD4^+^CD25^−^ T cells of CTRL, Hpx, and Hpx + T_4_ mice when cultured without anti-CD3 and anti-CD4 antibodies (Figure 6B). This result shows that CD4^+^CD25^−^ T cells of the offspring gestated in Hpx have a decreased differentiation capacity toward CD4^+^CD25^+^IL-10^+^FoxP3^+^ T cells.

### Adoptive Transfer of T CD4^+^CD25^+^ Cells Increase the Clinical Score and Onset of EAE in Mice Gestated in Hpx

**Figure 7:**
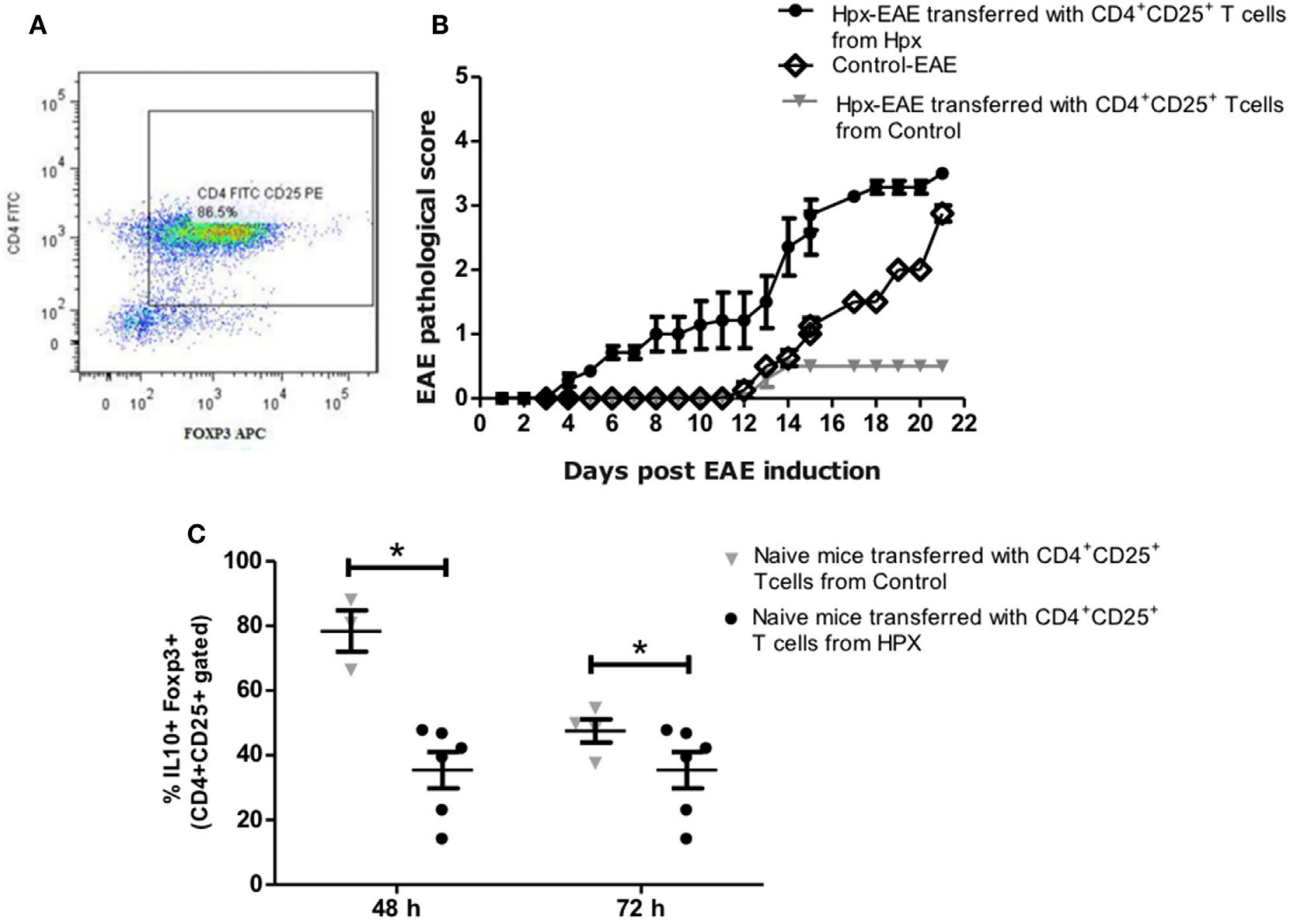
Adoptive transfer of CD4^+^CD25^+^ T cells derived from the offspring gestated in hypothyroxinemia (Hpx) increases the severity and onset of experimental autoimmune encephalomyelitis (EAE). **(A)** Representative dot-plot of the percentage of CD4^+^FOXP3^+^ T cells after CD4^+^CD25^+^ T cells were cultured *in vitro* for 7 days in the presence of IL-2 and anti-CD3 and anti CD8 antibodies. **(B)** The graph shows a pathological score of the recipient offspring gestated in Hpx after EAE induction (day 1) that received 1 × 10^6^ CD4^+^CD25^+^ T cells from mice gestated in euthyroidism (control) (inverted gray triangle) or Hpx (closed circle) that also suffer EAE. As control it shows also the pathological score of control offspring that were induced with EAE and did not receive CD4^+^CD25^+^ T cells (open diamond). *N* = 3 independent experiments. **(C)** The graph shows the quantification by flow cytometry of CD4^+^CD25^+^IL-10^+^ FOXP3^+^ T cells from splenocytes of naïve mice that previously were cultured *in vitro* for 48 or 72 h. These naïve mice were adoptively transfered with 1 × 10^6^ CD4^+^CD25^+^ T cells of the offspring gestated in Hpx (closed circles) or control (inverted gray triangles) that suffer EAE. The CD4^+^CD25^+^ T cells that were adoptively transfered are derived from spleen and were purified at day 21 of EAE induction in these mice and cultured *in vitro* for 7 days in the presence of IL-2 and anti-CD3 and anti-CD28 antibodies. Values represent the mean ± SEM. Statistical analysis was performed using one-way ANOVA and Tukey’s post-test. Statistical significance is indicated as *p < 0.05.

The role of suppression capacity during EAE disease was analyzed *in vivo* in the offspring gestated in Hpx. For that CD4^+^CD25^+^ T cells isolated from spleens of mice that suffer EAE were gestated in control or Hpx was kept *in vitro* for one week in the presence of IL-2 (2,000 IU/ml), anti-CD3 (1 µg/ml), and anti-CD28 (1 µg/ml). 1 × 10^6^ cells from these cell cultures were adoptively transferred into naive recipient mice gestated in Hpx. A representative histogram is shown in Figure 7A that shows the percentage of CD4^+^CD25^+^FOXP3^+^ T cells that were differentiated from CD4^+^CD25^+^ T cells from spleen of control mice (Figure 7A). Then 1 day after the cells were transferred, EAE was induced in these recipient mice. The pathological score was registered from day 1 after EAE induction (Figure 7B). Interestingly, mice that received CD4^+^CD25^+^ T cells that were activated *in vitro* and are derived from donors that were gestated in Hpx showed significantly higher EAE pathological score compared to recipient mice that received CD4^+^CD25^+^ T cells from control donors mice (Figure 7B). Interestingly, Hpx mice that received CD4^+^CD25^+^ T cells from control mice have less pathological score than control mice that did not receive these cells, suggesting that CD4^+^CD25^+^ T cells activated *in vitro* from control mice have the capacity to almost revert the effect of EAE induction. At 21 days after EAE induction the population of CD4^+^CD25^+^FOXP3^+^IL-10^+^ T cells was quantitated from the spleen of naïve recipient mice that received CD4^+^CD25^+^ T cells. These CD4^+^CD25^+^ T cells derived from mice gestated in Hpx or euthyroidism. The analysis of these results showed that there is a reduction in the content of CD4^+^CD25^+^FOXP3^+^IL-10^+^ T cells in those recipient mice that received CD4^+^CD25^+^ T cells from mice gestated in Hpx compared to those that received CD4^+^CD25^+^ T cells from mice gestated in euthyroidism (Figure 7C).

## Discussion

The knowledge about the effects of gestational Hpx has been oriented toward pregnancy outcome and the newborn neurological development (26). In this context, Hpx is associated with a 2.5-fold increase in the risk of preterm birth (27), an adverse early neonatal development (9), and an impaired child development in the long term (8, 28, 29). The impact of maternal THs specifically T_4_ deficiency over the immune system and the susceptibility to autoimmune diseases by the progeny is only the beginning to be elucidated (16). The first study to show that gestational hypothyroidism can influence the severity of EAE in the progeny was the study of Albornoz et al. (16). Here in this study, we provide new evidences supporting the notion that gestational T_4_ imprints the autoimmune response of the offspring. This work contributes with possible mechanisms involving the immune system that could enhance the susceptibility of the progeny to suffer an intense EAE. We observed that the offspring gestated in Hpx showed a significant increase in the EAE score at days 14 and 15 after EAE induction (Figure 2A); the offspring has high demyelination at day 21 after EAE induction (Figures 2B,C); and high infiltration of CD4^+^ and CD8^+^ T cells (Figures 2D–F) in spinal cords of mice after 21 days of EAE induction, as compared to mice gestated in euthyroidism. These results are consistent with the effects of gestational hypothyroidism over the outcome of EAE in the offspring (16). Given that both conditions, hypothyroidism and Hpx, have in common low T_4_ levels and these results reinforce the importance of appropriate levels of maternal T_4_ during pregnancy for the proper fetus development (30, 31). These results are consistent with previous observation of significant decrease in MBP protein expression in cortical tissue in the progeny gestated in Hpx or hypothyroidism at early stages after birth (postnatal day 16) (32). Thus, the demyelination observed in the adult offspring that was gestated in Hpx (Figures 2B,C) indicates that the gestational Hpx imprints the offspring until late adulthood. These observations correlate with other studies were the expression of myelin-related proteins reduced in the hippocampus in rats gestated in Hpx (5). Thus, gestational Hpx will not reach the necessary levels of T_4_ for the fetus. This might explain the reduced myelin content observed in the spinal cords from healthy and EAE suffering mice (Figures 2B,C).

In this study, spinal cord sections from the offspring gestated in Hpx had higher infiltration of CD4^+^ and CD8^+^ T cells compared to control (Figures 2D–F). Two hypotheses are proposed to search which are the mechanisms that change in the offspring gestated in Hpx that makes them more prone to infiltration and inflammation: (1) An alteration in the integrity of the blood–brain barrier of the offspring gestated in Hpx that will facilitate the migration of immune cells into the CNS. (2) The immune response could be strong in the progeny gestated in Hpx, this can be possible if T_Eff_ cells are overactive or T_reg_ cells are underactive. In this work, we focus in the population of CD4^+^CD25^+^ T cells and CD4^+^CD25^+^FOXP3^+^ T cells (considered to be part of T_reg_ cells classification), given that these cells play key roles in self-tolerance and autoimmunity (33). The T_reg_ cells play an inhibitory function over T_Eff_ cells by overcoming the activation of T_Eff_ cells in autoimmunity (24). In fact it has been shown that T_regs_ cells in MS patients have their regulatory function which is severely impaired (34). On the other hand, it has reported the alterations in the amount of T_reg_ in patients with MS (35). For example, it have been shown that the amount of CD25^+^ T_regs_ in cerebrospinal fluid diminished in patients with MS, but these cells remained similar at the peripheral blood (35, 36). However, the offspring gestated in Hpx had similar percentage of CD4^+^CD25^+^FOXP3^+^IL-10 T cells from spleen (Figure 3A) and CD4^+^FOXP3^+^ T cells and CD25^+^FOXP3^+^ T cells (Figures 4A,C,D) from spleen compared to mice gestated in euthyroidism. Moreover, after EAE induction the offspring gestated in Hpx has also similar percentage of CD4^+^FOXP3^+^ T cells and CD25^+^FOXP3^+^ T cells compared to the offspring gestated in euthyroidism (Figures 4B–D). Noteworthy is the fact that these cells were obtained after 21 days of EAE induction. It is possible that this is a late time point to detect differences in this T cells population. Thus, we propose for future studies to analyze CD4^+^CD25^+^FOXP3^+^IL-10 T cells population at early time points, when symptoms of EAE begin to appear in the mice suffering EAE. For example, at day 9 after EAE induction (Figure 2A) it will be possible to detect alterations in the population of CD4^+^CD25^+^FOXP3^+^ T cells. Even though, the offspring gestated in Hpx had similar percentage of CD4^+^CD25^+^FOXP3^+^IL-10^+^ T cells, several findings of this work suggest that these T cells have reduced capacity to suppress T_Eff_ cells. First, it is the observation that CD4^+^CD25^+^ T cells purified from spleen of naïve mice gestated in Hpx in *in vitro* suppression assays showed a reduced capacity to suppress the proliferation of T_Eff_ cell compared to the offspring gestated in control (Figure 5A). Second, CD4^+^CD25^−^ T cells purified from spleen of naïve mice and induced to differentiate in *in vitro* assays to T_reg_ showed less number of CD4^+^CD25^+^FOXP3^+^IL-10^+^ T cells compared to the offspring gestated in euthyroidism (Figure 6A). Third, adoptive transfer experiments of purified CD4^+^CD25^+^ T cells from the spleen of mice gestated in Hpx that suffer EAE, protected less (showed high pathological score) to the offspring gestated in Hpx that suffer EAE and received CD4^+^CD25^+^ T cells from the offspring gestated in euthyroidism and suffer EAE (Figure 7B). There are other results in this work that supports that the suppressive capacity of the immune system of the offspring gestated in Hpx is reduced: (1) Low content of IL-10 in the spleen of the offspring gestated in Hpx and that suffer EAE (Figures 4E,F); (2) the reduced secretion of IL-10 by CD4^+^CD25^−^ T cells exposed *in vitro* to a differentiation media for T_reg_ (Figure 6B); and (3) the low percentage of CD4^+^CD25^+^FOXP3^+^IL-10^+^ T cell population in the spleen of the recipient mice that suffer EAE and received CD4^+^CD25^+^ T cells from mice gestated in Hpx (Figure 7C). Even though, these results led us to propose the possibility that the offspring gestated in Hpx have a reduced capacity to induce suppression. We are not sure yet if these cell population are in fact T_reg_ cells given that it will be necessary to characterize other markers like CTLA-4, GITR, and CD127 (37). From this work, we could not conclude yet that the reduced capacity of CD4^+^CD25^+^ T cells from the offspring gestated in Hpx to suppress T_Eff_ cells is due to their lack of function, because we did not control if these cells could be suffering high rate of apoptosis or lack of proliferation (37). We observed, in this study, that CD4^+^CD25^+^ T cells from the spleen of the offspring gestated in Hpx have reduced capacity to differentiate *in vitro* to CD4^+^CD25^+^FOXP3^+^IL-10^+^ when they were stimulated with anti-CD3 and anti-CD28 antibodies (Figure 6A). If this phenotype can be extrapolated to an *in vivo* activation process (like in EAE) it could explain in part, why the offspring gestated in Hpx has an earlier onset and intense EAE than the offspring gestated in Hpx. In Figure 4B, we found a population of CD25^−^ T cells positive for FOXP3^+^. We have not characterized yet this population, however, it has been described that in humans with autoimmune diseases appear this population (38). We think that it is possible that gestational Hpx could be affecting T_reg_ cells function directly by diminishing its suppressive function. Among the many regulatory pathways described for T_regs_ the secretion of IL-10 is highlighted due to is inhibitory function in the production of cytokines, chemokines, prostaglandin E2, and antigen presentation, all involved in inflammation [reviewed by Moore et al. (39)]. The results of this work correlate with previous report about impaired secretion of IL-10 (40) and reduced suppression capacity of T_reg_ in MS patients (41). Thus, this works support the important role of T_reg_ in autoimmunity and this point is also observed in the experiments of T_reg_ adoptive transfer. T_reg_ from the offspring gestated in Hpx worsens the EAE pathological score of the recipient mice (Figure 7). However, it will be interesting to analyze the EAE phenotype and the immune response at earlier time points after EAE induction than day 21. Thus, the results from this work support that gestational Hpx diminished the capacity of the immune system of their offspring to suppress immune response. The new and old question is how THs can imprint the offspring. THs are known to act as transcription factors through their nuclear receptor (42, 43). Maternal T_4_ will mainly cross the placental barrier. These receptors are able to recruit cofactor complexes involved in chromatin remodeling/histone modifications in a T_3_ dependent manner (44). Thus, we propose that Hpx during pregnancy could cause a metabolic imprinting in the progenitors of fetus that will eventually affect their response when facing an autoimmune disease such as MS. Further experiments are required to assess how Hpx is affecting T_reg_ cell function, overall considering the environment in which T_reg_ cells execute their function during EAE and also their other suppressive mechanisms. The findings reported in this work support the notion that Hpx during pregnancy enhances the susceptibility of the progeny for suffering from EAE. Mice gestated in Hpx showed an early onset of the disease, increased demyelination, and impaired T_reg_ cell response. These data underscore the potential detrimental effects of maternal Hpx during pregnancy on the CNS and immune system function of their progeny.

## Ethics Statement

This study was carried out in accordance with the recommendations of CONICYT bioethical guidelines, and the Universidad Andres Bello bioethics committee. The protocol was approved by the Universidad Andres Bello bioethics committee.

## Author Contributions

HH and EA are both first authors of this manuscript; they equally contributed to this work. HH, EA, MO, and EJ have written the first draft of the manuscript. CR, SB, FS, AE, CC-V, and AK revised and improved the manuscript. MO, KB, LV, RB, TR-C and EJ contributed in the experiments and figures. All authors have seen and agreed on the finally submitted version of the manuscript.

## Conflict of Interest Statement

The authors declare that the research was conducted in the absence of any commercial or financial relationships that could be construed as a potential conflict of interest.

## Supplementary Material

### Complete Blood Count Analysis

Complete blood count (CBC) analysis was performed from blood samples obtained from the face vein of the offspring gestated in euthyroidism (control), Hpx or Hpx + T_4_ at P55 days old. The CBC analysis was performed by an external veterinary laboratory (VetLab) in Chile.

Figure S1 The offspring gestated in hypothyroxinemia (Hpx) weight the same than the offspring gestated in euthyroidism at the age of P55. The graph shows the weight of the offspring gestated in euthyroidism (control), Hpx, or Hpx + T4 at day P55 of age. The statistical analysis showed that there is not significant difference among the weights of the offspring gestated in Hpx, control, or Hpx + T_4_ mice. Control *N* = 28, Hpx *N* = 27, and Hpx + T_4_ *N* = 18. Mean ± SEM *p* ≤ 0.05, ANOVA, and Tukey’s test.

Figure S2 The weight of the offspring gestated in hypothyroxinemia (Hpx) during experimental autoimmune encephalomyelitis (EAE) induction behaves similar to the offspring gestated in euthyroidism. The graph shows the weight of the offspring gestated in euthyroidism (control), Hpx, or Hpx + T4 during the course of EAE induction. The weights were registered from day 0 (day before to EAE induction, mice had P55 days old), day 1 (day of EAE induction), and until day 21 (post-EAE induction). The analysis showed no significant differences between Hpx mice when compared to control and Hpx + T_4_. Control *N* = 18, Hpx *N* = 18, and Hpx + T_4_ *N* = 18. Mean ± SEM **p* < 0.05, ANOVA, and Tukey’s test.

Table S1 The offspring gestated in hypothyroxinemia (Hpx) has similar complete blood count (CBC) analysis compared to the offspring gestated in euthyroidism. A blood sample was taken at P55 days old of mice gestated in euthyroidism (control), Hpx, Hpx + T4 for CBC. The statistical analysis showed not significant differences between experimental groups. The offspring gestated in euthyroidism (control *N* = 5), in Hpx (*N* = 6), and Hpx + T_4_ (*N* = 3). Mean ± SEM **p* < 0.05, ANOVA, and Tukey’s test.

## Abbreviations

THs, thyroid hormones; THD, thyroid hormone deficiency; Hpx, hypothyroxinemia; EAE, experimental autoimmune encephalomyelitis; T_regs_, regulatory T cells; T_Eff_, effector T cells; MMI, methimazole; MS, multiple sclerosis; MOG, myelin oligodendrocyte glycoprotein; CNS, central nervous system; MBP, myelin basic protein.
