# Supplementary figures and images for: Gestational Hypothyroxinemia Affects Its Offspring With a Reduced Suppressive Capacity Impairing the Outcome of the Experimental Autoimmune Encephalomyelitis
Source: Front Immunol. 2018 Jun 6;9:1257. doi: 10.3389/fimmu.2018.01257 (PMC5997919; doi:10.3389/fimmu.2018.01257)

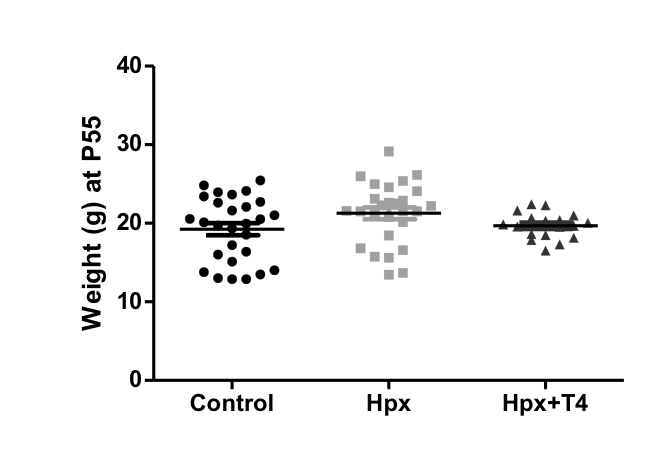

Supplement: Figure S1 — The offspring gestated in hypothyroxinemia (Hpx) weight the same than the offspring gestated in euthyroidism at the age of P55. The graph shows the weight of the offspring gestated in euthyroidism (control), Hpx, or Hpx + T4 at day P55 of age. The statistical analysis showed that there is not significant difference among the weights of the offspring gestated in Hpx, control, or Hpx + T4 mice. Control N = 28, Hpx N = 27, and Hpx + T4 N = 18. Mean ± SEM p ≤ 0.05, ANOVA, and Tukey’s test. [file image_1.jpeg]

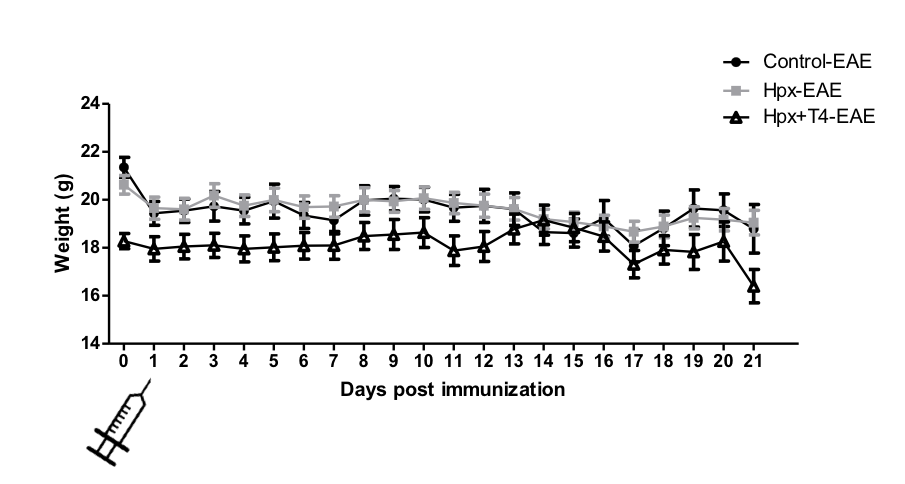

Supplement: Figure S2 — The weight of the offspring gestated in hypothyroxinemia (Hpx) during experimental autoimmune encephalomyelitis (EAE) induction behaves similar to the offspring gestated in euthyroidism. The graph shows the weight of the offspring gestated in euthyroidism (control), Hpx, or Hpx + T4 during the course of EAE induction. The weights were registered from day 0 (day before to EAE induction, mice had P55 days old), day 1 (day of EAE induction), and until day 21 (post-EAE induction). The analysis showed no significant differences between Hpx mice when compared to control and Hpx + T4. Control N = 18, Hpx N = 18, and Hpx + T4 N = 18. Mean ± SEM *p < 0.05, ANOVA, and Tukey’s test. [file image_2.jpeg]
