# Supplementary material for: Gestational Hypothyroxinemia Affects Its Offspring With a Reduced Suppressive Capacity Impairing the Outcome of the Experimental Autoimmune Encephalomyelitis
Source: Front Immunol. 2018 Jun 6;9:1257. doi: 10.3389/fimmu.2018.01257 (PMC5997919; doi:10.3389/fimmu.2018.01257)
Supplement: Table S1 — The offspring gestated in hypothyroxinemia (Hpx) has similar complete blood count (CBC) analysis compared to the offspring gestated in euthyroidism. A blood sample was taken at P55 days old of mice gestated in euthyroidism (control), Hpx, Hpx + T4 for CBC. The statistical analysis showed not significant differences between experimental groups. The offspring gestated in euthyroidism (control N = 5), in Hpx (N = 6), and Hpx + T4 (N = 3). Mean ± SEM *p < 0.05, ANOVA, and Tukey’s test. [file table_1.docx]

**Supplementary table I: CBC analysis**

|  | **Units** | **Control** | **Hpx** | **Hpx+T_4_** |
| --- | --- | --- | --- | --- |
| **Red blood cell count (RBC)** | X10^6 cells/µL | 8.654±0.146 | 8.277±0.319 | 8.210±1.343 |
| Hematocrit | % | 41.02±0.809 | 39.97±1.206 | 37.00±1.414 |
| Hemoglobin | g/dL | 13.02±0.244 | 13.37±0.528 | 12.30±0.400 |
| Mean cell volume (MCV) | fL | 47.40±0.685 | 48.38±0.825 | 46.67±5.207 |
| Mean cell hemoglobin (MCH) | pg/cell | 15.80±0.143 | 16.15±0.278 | 15.63±1.963 |
| Mean cell hemoglobin concentration (MCHC) | g/dL | 31.84±0.359 | 33.38±0.669 | 33.33±0.667 |
| Percent reticulocytes | % | 1.2±0.127 | 1.67±0.329 | 1.133±0.066 |
| Reticulocyte count | X10^3 cells /uL | 103.7±10.96 | 136.7±25.68 | 94.37±19.38 |
| Platelet count | X10^3 cells /uL | 478.2±112.6 | 509.8±90.23 | 310±85.05 |
| **White blood cell count (WBC)** | X10^3 cells /µL | 9±0.709 | 8.45±0.538 | 8.6±1.002 |
| Percent neutrophils | % | 38.60±2.731 | 32.50±5.841 | 31.±3.786 |
| Percent Lymphocytes | % | 56±2.915 | 62±6.116 | 64±4.163 |
| Percent Monocytes | % | 3.2±0.581 | 4±0.447 | 4±0.577 |
| Percent Eosinophil | % | 2.2±0.374 | 1.5±0.342 | 1±0.0 |
| Neutrophil count | /µL | 3495±414.5 | 2831±585.5 | 2590±35.18 |
| Lymphocyte count | /µL | 5018±446.6 | 5156±463.5 | 5587±1025 |
| Monocyte count | /µL | 283±51.73 | 332.8±187.6 | 336.3±37.9 |
| Eosinophil count | /µL | 204.2±42.02 | 344±187.6 | 86±10.02 |
